# The Autophagy–Inflammation Axis in Kawasaki Disease: Pathogenic Mechanisms and Translational Opportunities

**DOI:** 10.3390/jcm15103918

**Published:** 2026-05-19

**Authors:** Qian Xu, Yali Wu, Yan Ding

**Affiliations:** Department of Rheumatology and Immunology, Clinical Research Center of Pediatric Rheumatic and Immunological Diseases, Institute of Maternal and Child Health, Wuhan Children’s Hospital (Wuhan Maternal and Child Health Care Hospital), Tongji Medical College, Huazhong University of Science & Technoogy, Wuhan 430074, China; 17815370932@163.com (Q.X.);

**Keywords:** Kawasaki disease, autophagy–inflammation axis, vasculopathy, NLRP3/cGAS-STING pathway, therapeutic targets

## Abstract

Kawasaki disease (KD) represents the foremost cause of acquired pediatric heart disease, with coronary artery injury being the principal factor contributing to adverse prognoses. A significant clinical challenge is that 20–30% of patients demonstrate resistance to intravenous immunoglobulin (IVIG), which markedly elevates the risk of coronary artery lesions and long-term cardiovascular sequelae. Consequently, there is an urgent need to investigate novel pathogenic mechanisms beyond the conventional cytokine storm theory and to identify effective therapeutic targets. This review systematically summarizes the key role of the autophagy–inflammation axis in KD vasculopathy. Current evidence indicates that defective mitophagy and lysosomal dysfunction induce mitochondrial DNA release, resulting in overactivation of the NLRP3 inflammasome and cGAS-STING pathways, which amplify inflammatory responses and aggravate endothelial damage. The regulation of this axis is dynamic during both the acute and recovery phases and is influenced by metabolic reprogramming and epigenetic modifications, which may partially explain the lack of response to IVIG. Pharmacological agents, such as rapamycin and metformin, as well as natural compounds, such as resveratrol and urolithin A, have demonstrated beneficial anti-inflammatory effects in preclinical studies. Targeting the autophagy–inflammation axis represents a significant research direction with the potential to evolve into a promising therapeutic strategy. Mechanistically, restoring the balance of the autophagy–inflammation axis holds promise for mitigating coronary complications and improving long-term cardiovascular outcomes in children with KD; however, this prospect requires validation through prospective clinical studies.

## 1. Introduction

Kawasaki disease (KD) is an acute systemic vasculitis that predominantly affects infants and young children. The most concerning complication is coronary artery injury, which is the leading cause of acquired heart disease in children. Although intravenous immunoglobulin combined with aspirin has become the standard therapeutic regimen, approximately 20–30% of affected children fail to respond to the initial treatment, with a significantly elevated risk of coronary artery lesions. This clinical dilemma necessitates an in-depth exploration of the deeper pathological mechanisms that extend beyond the conventional “cytokine storm” framework [1,2].

In recent years, the dual role of autophagy in immune and inflammatory diseases has attracted increasing scholarly attention. Autophagy contributes to the maintenance of internal environmental homeostasis by eliminating damaged organelles and invading pathogens. Conversely, dysregulated autophagy can intensify aberrant amplification of inflammatory signalling. In the context of Kawasaki disease research, a noteworthy observation is the significant downregulation of key autophagy markers (e.g., ATG16L1 and LC3II) in the peripheral blood of IVIG non-responders. This downregulation is positively correlated with an elevated risk of coronary artery aneurysm development [3,4]. This evidence suggests that the functional status of autophagy may not merely be a passive element in the disease process but rather a regulatory factor influencing disease prognosis.

Nevertheless, current studies have mostly focused on autophagy or inflammation at the individual level rather than their interplay. The manner in which these two processes interact and intertwine to form an axis network that drives vascular injury, as well as the patterns governing their systemic integration and dynamic evolution, remain poorly understood [5].

Furthermore, the application of single-cell multi-omics technologies has enabled the detailed delineation of imbalanced T/B-cell subsets and aberrant activation of myeloid cells in Kawasaki disease vasculitis. However, the “crosstalk” between these immune cell abnormalities and the autophagic status of endothelial cells to collectively shape the vasculitic microenvironment remains an unsolved enigma [6]. This study aimed to systematically dissect the core role of the autophagy–inflammation axis in vascular injury associated with Kawasaki disease.

Therefore, this review seeks to offer a comprehensive synthesis and critical evaluation of the autophagy–inflammation axis in Kawasaki disease (KD) vasculopathy. Unlike previous reviews that primarily address the general immunopathogenesis of KD, the novelty of this work resides in its integrative framework, which specifically connects defective mitophagy and lysosomal dysfunction to the overactivation of the NLRP3/cGAS-STING pathways. It further investigates their roles in intravenous immunoglobulin (IVIG) non-response and assesses emerging translational therapeutic strategies within this context. Initially, we will elucidate the molecular underpinnings of this axis by concentrating on how mitophagy defects and lysosomal dysfunction trigger inflammatory cascades via the NLRP3/cGAS-STING pathways. Subsequently, we will examine its dynamic progression across the acute and convalescent stages and analyse its interactions with immune dysregulation and clinical heterogeneity. Furthermore, we will explore multilevel regulatory networks, including metabolic reprogramming and epigenetic modifications, that influence this axis. Finally, we will assess emerging therapeutic strategies targeting this axis, discuss ongoing translational challenges, and propose a framework for integrating multi-omics data to facilitate risk-stratified management and precision therapeutics, ultimately aiming to enhance long-term cardiovascular outcomes in children with KD [7,8].

### Methods of the Review

This narrative review synthesizes the current evidence on the role of the autophagy–inflammation axis in Kawasaki disease (KD) vasculopathy. A comprehensive literature search was performed in the PubMed/MEDLINE, Web of Science, and Scopus databases for articles published between January 2000 and March 2026. The search utilized a combination of the following keywords and their variants: “Kawasaki disease,” “autophagy,” “mitophagy,” “lysosome,” “NLRP3 inflammasome,” “cGAS-STING,” “vascular inflammation,” “coronary artery lesion,” “IVIG resistance,” and “therapy.” Original research articles and relevant reviews were considered. Studies were prioritised based on their direct relevance to the mechanistic interplay between autophagy and inflammation in KD, focusing on human studies, established KD animal models (e.g., LCWE and CAWS), and in vitro models using KD patient serum or relevant endothelial/immune cells. Evidence extrapolated from broader cardiovascular or inflammatory contexts has been explicitly noted. The synthesis aimed to construct a unified framework linking mitochondrial/lysosomal homeostasis, inflammatory signalling, clinical heterogeneity, and therapeutic implications.

## 2. Molecular Mechanisms of the Autophagy–Inflammation Axis

### 2.1. Mitophagy Abnormalities and Inflammatory Signaling Activation

Transcriptomic analysis by Lin et al. revealed that minichromosome maintenance complex component 8 (MCM8) gene expression was significantly downregulated in the peripheral blood of patients with KD who had coronary artery aneurysms, accompanied by upregulation of type I interferon signalling pathways. This study demonstrated that coronary artery inflammation was significantly aggravated in Mcm8 gene knockout mouse models, with disorganised mitochondrial arrangement and reduced mitochondrial numbers in cardiomyocytes, and showed that the cGAS-stimulator of interferon genes (STING)-type I interferon pathway was aberrantly activated. The primary mechanism involves the inhibition of nitric oxide (NO) synthesis, which disrupts E3 ubiquitin (parkin) ligase-mediated MCM8 ubiquitination modification, inhibits the dissociation of the MCM8-MCM9 complex, consequently interferes with MCM8 mitochondrial localisation, and ultimately impairs mitophagy function. Specifically, suppressed NO synthesis attenuates parkin ubiquitination, resulting in ineffective ubiquitin-mediated tagging of MCM8 and subsequent failure to initiate mitophagy, ultimately leading to mitochondrial accumulation. Notably, the MCM8-P276 genetic variant, which is specific to East Asian populations, can lead to reduced mitophagy capacity, and this variant is significantly associated with KD susceptibility [9]. Together, these findings from a murine model suggest that targeting MCM8 or the cGAS-STING pathway could be explored as a potential direction for precision therapy in KD, particularly in light of the association between the East Asian-enriched MCM8-P276 variant and KD susceptibility [9].

Furthermore, evidence extrapolated from broader cardiovascular or inflammatory contexts suggests that downregulation of peroxisome proliferator-activated receptor gamma coactivator 1-alpha (PGC-1α) expression can impair parkin-dependent mitophagy [5,10]. It is important to note that the specific role of PGC-1α in KD and its association with IVIG resistance remain to be validated in KD-specific models.

### 2.2. Lysosomal Homeostasis Imbalance and Inflammasome Activation

West et al. demonstrated that mitochondrial transcription factor A (TFAM) is responsible for packaging and stabilising mtDNA. TFAM deficiency can lead to abnormal mtDNA packaging, resulting in leakage due to packaging defects, which subsequently triggers type I interferon responses through the cGAS-STING-IRF3 axis. The fusion of lysosomes with autophagosomes to form mitolysosomes is a critical step in clearing damaged mitochondria. If lysosomal function is impaired (such as in age-related lysosomal acidification disorders), damaged mitochondria accumulate, significantly aggravating mtDNA leakage and thereby markedly activating inflammasomes. In related disease models such as aging, the core mechanism underlying impaired lysosomal acidification involves reduced activity of the proton pump (v-ATPase) on the lysosomal membrane, leading to elevated intralysosomal pH [11]. Whether a similar lysosomal acidification dysfunction exists in IVIG non-responders with KD is an important hypothesis for future validation.

Furthermore, research conducted by Marek-Iannucci et al. has demonstrated that small molecule compounds, such as urolithin A (uroA), can mitigate mtDNA leakage by enhancing mitophagy, thereby inhibiting the activation of the cGAS-STING signalling pathway. This finding suggests that the regulation of lysosomal function is a critical factor in maintaining the balance between autophagy and inflammation [5,12]. In the context of KD, it is hypothesised that lysosomal dysfunction may contribute to obstructed autophagic flux and uncontrolled inflammatory responses, potentially exacerbating vascular damage [5,11]. This hypothesis is supported by studies on related inflammatory conditions, which indicate that lysosomal impairment can amplify NLRP3 inflammasome activation [11].

### 2.3. Synergistic Effects of Mitophagy Defects and Lysosomal Dysfunction

Previous research has demonstrated that defects in mitophagy result in substantial accumulation of damaged mitochondria, thereby significantly increasing the burden on lysosomal clearance. Conversely, disorders in lysosomal acidification or degradation can obstruct the final step of mitophagy, that is, fusion with the lysosomes. This creates a detrimental cycle of “autophagy defect and increased lysosomal load,” which further facilitates mtDNA leakage, persistently overactivates the NLRP3/cGAS-STING pathway, markedly amplifies pro-inflammatory cytokine release signals, and ultimately exacerbates vascular endothelial inflammation and structural damage. This process plays a critical role in the progression of coronary artery lesions in patients with Kawasaki disease. This vicious cycle is most pronounced during the acute phase of Kawasaki disease (1–2 weeks post-onset), when mitophagy defects are most severe, lysosomal load reaches its peak, and mtDNA leakage escalates in tandem with pro-inflammatory cytokine levels. Upon entering the convalescent phase (≥4 weeks post-onset), this cycle gradually diminishes as autophagic activity partially recovers [5,10].

### 2.4. Specific Regulation of Inflammatory Responses by Selective Autophagy

Kimura et al. recently revealed that selective autophagy participates in the precise regulation of KD vascular inflammation by targeting the degradation of key inflammatory regulatory molecules. Specifically, NOD1 selectively degrades apoptosis signal-regulating kinase 1 (ASK1) through an autophagy-dependent pathway involving ATG16L1, blocking excessive activation of the endoplasmic reticulum stress-ASK1-p38 MAPK pathway, and thereby inhibiting coronary artery inflammation. The study found that in NOD1-deficient mouse models, coronary artery inflammation scores were 70% higher than those in wild-type mice, further confirming the anti-inflammatory role of this pathway in KD [13].

**Figure 1:**
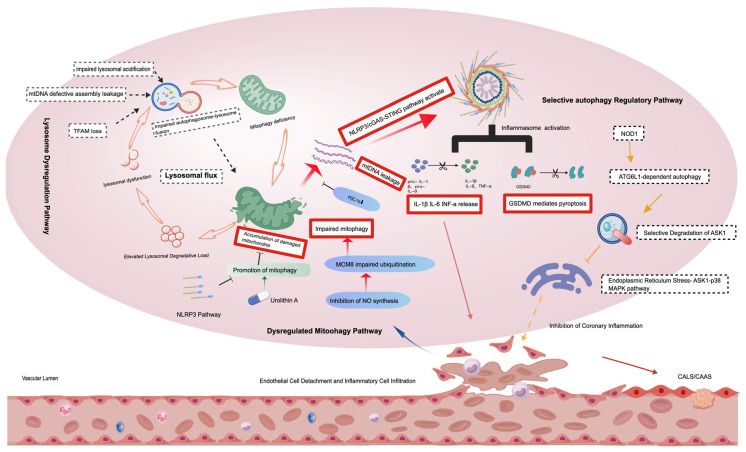
Schematic of the dysregulated autophagy–inflammation axis in Kawasaki disease vasculopathy. This schematic illustrates the core hypothesis linking defective autophagy to excessive inflammation in KD vascular injury. The central, highlighted pathway (bold arrows and boxes) depicts the key sequence: impaired mitochondrial autophagy (mitophagy) leads to the accumulation of damaged mitochondria and cytosolic release of mitochondrial DNA (mtDNA). Cytosolic mtDNA is sensed by the cGAS-STING pathway, which activates the NLRP3 inflammasome. Inflammasome activation triggers gasdermin D (GSDMD)-mediated pyroptosis and the maturation/release of pro-inflammatory cytokines (e.g., IL-1β, IL-18), driving endothelial dysfunction and coronary artery lesions (CALs). Upstream regulatory factors contributing to autophagic flux inhibition, such as lysosomal dysfunction and endoplasmic reticulum (ER) stress activating the ASK1-p38 MAPK pathway, are grouped in light-grey dashed boxes to indicate their modulatory role. Potential therapeutic intervention points are indicated (e.g., Urolithin A, Necrosulfonamide). The arrow styles denote the level of evidence: solid arrows represent pathways with stronger support from KD-specific studies; dashed arrows represent more hypothetical or extrapolated connections. Abbreviations: CALs, Coronary Artery Lesions; cGAS, cyclic GMP-AMP synthase; GSDMD, gasdermin D; IL, interleukin; mtDNA, mitochondrial DNA; STING, stimulator of interferon genes.

Additionally, Han et al. and Jia et al. demonstrated that KD patient serum can induce pyroptosis in macrophages within human umbilical vein endothelial cells (HUVECs), thereby triggering excessive NLRP3 inflammasome activation, promoting caspase-1 cleavage and maturation, and IL-1β release. The GSDMD inhibitor, necrosulfamide, can reverse this effect and alleviate vascular inflammation. In vitro assays showed that 10 μmol/L necrosulfamide treatment significantly diminished the macrophage pyroptosis rate from 68.3% to 21.7%, concomitant with a 72.5% reduction in IL-1β release (*p* < 0.01) [14,15]. Consequently, it can be hypothesized that pyroptosis in macrophages and vascular endothelial cells (mediated by GSDMD) may be associated with autophagy defects. The core mechanism is shown in Figure 1.

Although the studies summarized above implicate both autophagic defects (e.g., downregulation of MCM8 and PGC-1α) and inflammatory hyperactivation (e.g., NLRP3/cGAS-STING) in KD vasculopathy, direct evidence causally linking them into a coherent “axis” in vivo remains incomplete. For instance, the work by Kimura et al. on the NOD1-ASK1 pathway [13] and by Han et al. on GSDMD-mediated pyroptosis [14,15] provide supportive pieces from the perspectives of “autophagy regulating inflammation” and “inflammatory execution causing damage,” respectively. However, their spatiotemporal correlation within the lesional vasculature of KD patients and how they are co-regulated by upstream metabolic or epigenetic cues lack systematic investigation. Future research employing spatiotemporal multi-omics on the same KD model or patient samples is needed to simultaneously map autophagic flux and inflammatory signaling, thereby validating the integrity and dynamic interplay of this proposed axis.

## 3. Dynamic Imbalance Characteristics of the Autophagy–Inflammation Axis in Kawasaki Disease

### 3.1. Pathophysiological Changes in Acute and Recovery Phases

Numerous studies have indicated that significant mitophagy dysfunction may be the core characteristic of the acute phase of KD, primarily manifested by downregulation of mitophagy regulatory factors, such as PGC-1α and MCM8, leading to accumulation of damaged mitochondria and massive release of mtDNA, which subsequently triggers systemic inflammatory responses through activation of the cGAS-STING and NLRP3 pathways. However, autophagy activity in patients with KD gradually recovers during the recovery phase, allowing partial recovery of mitochondrial function. Although peripheral blood inflammatory factor levels significantly decrease in patients, persistent chronic low-grade inflammation remains, which is thought to further induce abnormal proliferation of vascular smooth muscle cells and collagen deposition, a process implicated in the formation of coronary artery aneurysms (CAAs) and vascular remodeling [5,9,10]. The severity and persistence of this axis imbalance during the acute phase may correlate with the risk of developing CAAs and resistance to intravenous immunoglobulin (IVIG) therapy [5,16].

Therefore, profiling the activity of this axis (e.g., via markers such as LC3B, MCM8, or mtDNA) could help distinguish between IVIG-resistant and IVIG-responsive patients and predict the risk of progressive coronary artery lesions.

### 3.2. Clinical and Experimental Evidence Support

Wang et al. found that MCP-1 levels in peripheral blood mononuclear cells of KD patients during the acute phase showed a significant negative correlation with the autophagy marker LC3B expression (*p* < 0.01). Moreover, the decrease in LC3B expression was significantly greater in KD patients with coronary artery aneurysms compared to those without coronary artery involvement [16]. The mechanism underlying this phenomenon primarily involves MCP-1 inhibiting nuclear translocation of transcription factor EB (TFEB) through CCR2 receptor-dependent pathways, leading to reduced expression of lysosomal biogenesis key protein LAMP1, and ultimately impeding the autophagosome-lysosome fusion process, mainly manifested as an increased LC3B-II/LC3B-I ratio [17].

Studies have shown that the classical inflammasome NLRP3-related pathway plays a critical role in the pathological processes of KD in mouse models and patients during the acute phase [15,18,19]. In a KD mouse model induced by Lactobacillus casei cell wall extract (LCWE), single-cell transcriptomic analysis revealed significant myeloid cell infiltration at coronary artery lesion sites, with high expression of NLRP3, IL-1β, and IL-18 in the infiltrating myeloid cells. Spatial transcriptomics further confirmed that these inflammatory cells formed specific spatial co-localisation patterns with vascular smooth muscle cells and fibroblasts, promoting vascular remodelling through paracrine signaling [20]. Additionally, Na et al. mentioned in their study that neutralising antibody intervention against the NLRP3 inflammasome could reduce arterial inflammation scores [21], further suggesting that targeting inflammasomes represents an effective intervention strategy for KD vascular inflammation.

Zhang et al. discovered that TIMD4 + MHC II + macrophages accumulating in the heart can delay heart failure through RETNα (RELMa)-mediated regulation of autophagic flux. The removal of this cell subset or knockout of RETNα accelerates cardiac functional damage in mice [22]. Furthermore, some studies have shown that resistin secreted by bone marrow-derived monocytes or macrophages can promote the release of IL-6, TNF-α, and other inflammatory factors through activation of the NF-κB pathway, further inducing vascular endothelial dysfunction and proliferation of vascular smooth muscle cells, participating in the development of atherosclerosis, coronary artery disease, and other pathological conditions [23,24]. Consequently, it is hypothesised that resistin may exacerbate vascular inflammation by inhibiting autophagic flux. Its abnormal expression is therefore a candidate biomarker, suggesting that it may be associated with the severity of coronary artery lesions in patients with KD, a notion that requires validation in clinical cohorts. In vitro studies demonstrated that treatment with recombinant resistin attenuated the LC3B-II/LC3B-I ratio in vascular endothelial cells from 1.8 to 0.7 and diminished nuclear translocation of TFEB by 63.4% (*p* < 0.01), thereby corroborating the suppressive effect of resistin on autophagic flux.

**Figure 2:**
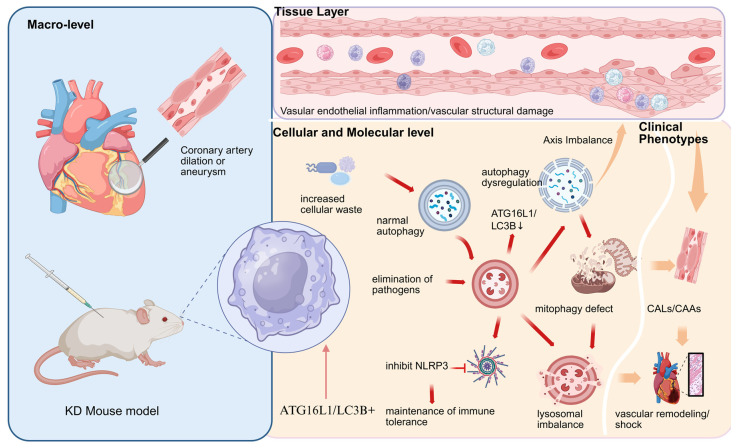
Framework of the Autophagy–Inflammation Axis and Coronary Artery Injury in Kawasaki Disease. This schematic delineates the pathogenic cascade from systemic manifestations to cellular dysfunction and clinical outcomes. Macro Level: The overview depicts the heart and coronary arteries, situating the disease in its anatomical context. The inclusion of a Kawasaki disease (KD) mouse model indicates the experimental system used for mechanistic validation. Tissue Level: Focal vascular endothelial inflammation and structural damage are highlighted as the primary tissue-level events. Cellular and Molecular Level: This core section illustrates the dysregulated autophagy–inflammation axis. Key processes include fluctuations in autophagy-related proteins (ATG16L1 and microtubule-associated protein 1 light chain 3 B, LC3B), accumulation of cellular waste, and a deficit in mitophagy. The inhibition of the NOD-like receptor family pyrin domain containing 3 (NLRP3) inflammasome is shown as a regulatory mechanism within this axis, contributing to the maintenance of immune tolerance under physiological conditions. Imbalances in this axis disrupt immune homeostasis. Clinical Phenotypes: Persistent molecular and cellular dysregulation culminates in the defining clinical phenotypes of KD: coronary artery lesions (CALs), coronary artery aneurysms (CAAs), vascular remodeling, and systemic inflammatory shock. Arrows indicate proposed directional relationships or influences between components across levels.

Numerous studies have also found that CD19+ B cell counts are significantly elevated in KD patients during the acute phase and return to normal after IVIG treatment, suggesting that abnormal B cell activation may enhance autophagy defect-mediated vascular inflammation through the secretion of inflammatory factors, forming an “immune cell activation–autophagy disorder–inflammation amplification” regulatory network [7,15,17,21], further regulating the occurrence of KD. Collectively, this evidence sketches a complex network of “immune cell–endothelial cell” interactions in which inflammatory factors from monocytes/macrophages (e.g., MCP-1, resistin) suppress endothelial autophagy, whereas autophagic defects and pyroptosis in endothelial cells release damage signals that further recruit and activate immune cells, forming a vicious cycle. Aberrantly activated CD19+ B cells may act as amplifiers in this cycle. However, a significant knowledge gap exists in determining the primary initiating event in vivo: does immune cell dysregulation precede and cause endothelial autophagic failure, or does an intrinsic endothelial autophagic defect trigger the abnormal immune response? Clarifying this sequence of causality is crucial for identifying optimal therapeutic targets. The autophagy–inflammation axis and coronary artery injury in Kawasaki disease are shown in Figure 2.

## 4. Multi-Level Regulatory Network of the Autophagy–Inflammation Axis

### 4.1. Metabolic Reprogramming Regulation

Salminen et al. demonstrated that AMPK is a cellular energy sensor that coordinates the balance between autophagy and inflammation through multiple mechanisms: (1) directly phosphorylating ULK1 (Ser317 site) to activate autophagy initiation; (2) increasing NAD+ levels to activate SIRT1, which then inhibits NF-κB p65 (Lys310) transcriptional activity through deacetylation, thereby reducing NF-κB target gene reporter activity; and (3) promoting PGC-1α-mediated mitochondrial biogenesis, further reducing reactive oxygen species (ROS) accumulation [25].

Additionally, studies have shown that glutamine metabolism exhibits dynamic biphasic regulatory characteristics for the autophagy–inflammation axis. Short-term starvation can activate autophagy by inhibiting the mTOR pathway, whereas long-term starvation replenishes the lysosomal pool through autophagic lysosome reformation mechanisms [26], maintaining autophagy homeostasis. Its downstream metabolite glutathione (GSH) not only affects T cell autophagy by regulating mTOR signalling but also reduces NLRP3 inflammasome activation levels through antioxidant effects [27]. Wang et al. also found that KD patients exhibit abnormalities in bile acid and lipid metabolism, and lipid metabolic abnormalities can inhibit autophagic flux by elevating ROS and activating the mTOR pathway, further exacerbating inflammatory responses, which can be partially reversed by IVIG treatment [28]. The aforementioned studies suggest that related metabolic regulation of autophagy may promote the occurrence of KD.

### 4.2. Epigenetic Modification Regulation

Qiao et al. demonstrated that DNA methyltransferase DNMT3b is activated under oxidative stress conditions, leading to increased methylation levels in CpG islands of the ATG7 promoter region, significantly inhibiting autophagic flux [29]. Conversely, histone deacetylase inhibitors (such as TSA) can promote TFEB nuclear translocation through increased H3K9ac modification, upregulating lysosomal gene expression. Studies have found synergistic regulatory effects between DNA demethylation and histone acetylation, where TET2-mediated DNA demethylation can recruit p300 acetyltransferase to co-activate autophagy-related gene transcription.

Furthermore, single-cell sequencing studies have found that CD14+ monocyte numbers are significantly increased in the early stages of KD vasculitis, forming specific co-expression modules with CD19+ B cells that participate in immune response regulation. CD14+ monocytes mediate communication with other immune cells through signalling molecules, such as Selplg and Itk [30]. The activity of these immune communication modules may differ between patients with complete versus incomplete KD or those with varying risks of CAL development, representing a link between epigenetic immunological regulation and clinical heterogeneity.

### 4.3. Pathogen-Associated Molecular Pattern Activation

**Figure 3:**
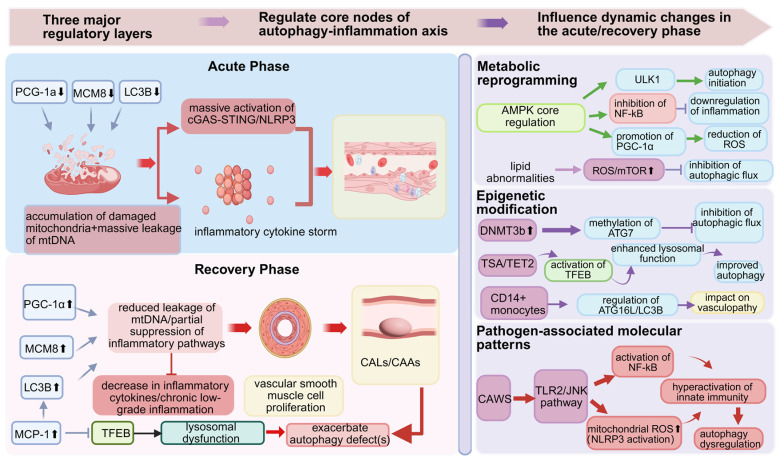
Dynamic Imbalance of the Autophagy–inflammation Axis Across Acute and Recovery Phases and Its Multilayer Regulatory Network. The schematic illustrates the stage-specific dysregulation of core cellular processes and external regulatory inputs that influence the axis. Three major regulatory layers (**top**) modulate the axis: (1) metabolic reprogramming (e.g., via AMPK, mTOR, and ROS), (2) epigenetic modification, and (3) pathogen-associated molecular pattern (PAMP) recognition (e.g., via NF-κB). The acute phase (**left**) is characterized by the downregulation of key regulators—PGC-1α, MCM8, and the autophagic marker LC3B—leading to the accumulation of damaged mitochondria and massive mitochondrial DNA (mtDNA) leakage. This triggers robust activation of the cGAS–STING and NLRP3 inflammasome pathways. The recovery phase (**right**) shows partial recovery, with upregulation of PGC-1α, MCM8, and LC3B, reduced mtDNA leakage, and dampened inflammatory signaling. However, persistent lysosomal dysfunction, elevated MCP-1, and altered TFEB activity contribute to chronic low-grade inflammation, vascular smooth muscle cell proliferation, and apoptotic/neuroprotective alterations. The bottom section integrates how the imbalanced axis influences vascular pathology, including smooth muscle remodeling and monocyte (CD14+) involvement. Arrows indicate proposed activating or inhibitory relationships.

Studies have shown that pathogen-associated molecular pattern mimetic Candida albicans cell wall extract (CAWS) can dual-regulate immune responses through the TLR2/Dectin-2-Syk-JNK pathway. On one hand, it activates the NF-κB pathway to promote secretion of pro-inflammatory factors such as IL-6, elevating IL-6 levels; on the other hand, it induces mitochondrial ROS burst, thereby activating the NLRP3 inflammasome3. Another study demonstrated that CAWS can induce coronary arteritis in T/B cell-deficient mice, confirming that its effects depend on innate immune system hyperactivation rather than adaptive immune responses, For CAWS-induced modeling, mice received intraperitoneal injections of CAWS (2 mg per animal) daily for 5 consecutive days. Overt coronary arteritis subsequently developed within 7–14 days post-modeling, with a mean inflammatory score of 3.2 ± 0.42. These findings suggest that CAWS may regulate autophagy through the aforementioned pathways, which could represent a mechanism contributing to coronary artery damage in experimental models. A regulatory network diagram is shown in Figure 3.

## 5. Therapeutic Strategies Targeting the Autophagy–Inflammation Axis

### 5.1. Current Standard of Care and Unmet Need

The initial management of Kawasaki disease relies on high-dose intravenous immunoglobulin (IVIG) combined with aspirin, which effectively resolves inflammation and prevents coronary artery lesions (CALs) in approximately 70–80% of patients [1,2]. For patients with IVIG-resistant disease, second-line options include corticosteroids, the anti-TNFα monoclonal antibody infliximab, and calcineurin inhibitors such as cyclosporine [1,2]. Despite this regimen, a subset of patients still develops CALs, and the optimal management of refractory disease remains challenging. This unmet clinical need drives the investigation of therapies targeting fundamental pathogenic mechanisms, such as the autophagy–inflammation axis, which may serve as adjunctive, rescue, or preventative strategies.

### 5.2. A Framework for Evaluating Novel Therapies

The following sections critically evaluate potential therapeutic agents and targets within the autophagy–inflammation axis. Interventions are categorized based on their level of evidence and translational readiness (Table 1). The evaluation for each agent considers the following: (i) the source and strength of evidence (from human KD studies to extrapolation from other fields), (ii) the proposed therapeutic objective (e.g., prevention of CALs, treatment of IVIG-resistant disease), (iii) pediatric relevance and safety considerations, and (iv) major limitations for clinical development.

### 5.3. Clinically Applied and Near-Term Candidates

#### Anakinra (IL-1 Receptor Antagonist)

Anakinra, an IL-1 receptor antagonist, directly inhibits the output of the NLRP3 inflammasome, a core effector of the autophagy–inflammation axis. Its therapeutic rationale is supported by human genetic studies linking IL1B polymorphisms to IVIG resistance [31] and clinical case series reporting efficacy in refractory KD [32,33], positioning it as a rescue therapy for IVIG-resistant cases. It represents the most immediate candidate for clinical translation because of its precise mechanism and established pediatric safety profile. However, its requirement for subcutaneous administration, high cost, and the need for larger controlled trials to define optimal dosing are practical limitations. Nonetheless, it remains the prime near-term candidate for evaluating IL-1 blockade in severe KD.

### 5.4. Preclinical Candidates with KD-Specific Evidence

#### 5.4.1. Sirolimus (Rapamycin)

Rapamycin, a classical mTOR inhibitor, has demonstrated efficacy in preventing coronary artery remodeling in a murine model of Kawasaki disease [34], underscoring its potential as a disease-modifying therapy. This in vivo evidence positions it as a candidate for intervening in the pathological vascular restructuring that underlies coronary complications. Although its mechanism of autophagy induction is well-characterized and its immunosuppressive properties are utilized in other pediatric conditions, its clinical translation for acute KD faces significant challenges due to a narrow therapeutic window, potent immunosuppressive effects, and unresolved questions regarding optimal timing during the acute inflammatory phase.

#### 5.4.2. Necrosulfonamide (GSDMD Inhibitor)

Necrosulfonamide inhibits pore-forming protein gasdermin D (GSDMD), a key executor of pyroptosis downstream of inflammasomes. In vitro studies have shown that it significantly reduces macrophage pyroptosis and IL-1β release induced by KD patient serum [14,15], supporting its potential as a rescue therapy for mitigating acute endothelial inflammation. Although it targets a pathway directly implicated in KD models, its specificity, pharmacokinetics, and in vivo efficacy require thorough validation, and clinical-grade inhibitors for systemic use are not yet available, relegating it to the early preclinical stage.

#### 5.4.3. Resveratrol

The natural polyphenol resveratrol exerts anti-inflammatory effects associated with autophagy induction. KD-specific evidence includes its ability to alleviate myocardial injury in a mouse model [35] and induce autophagy in human coronary arterial endothelial cells in vitro [36], supporting its role as a potential adjunctive antioxidant and anti-inflammatory agent. Its favourable safety profile is advantageous; however, its extremely poor oral bioavailability and rapid metabolism, coupled with a lack of controlled clinical trials in KD, severely limit its current clinical utility unless advanced, bioavailable formulations are developed.

### 5.5. Mechanistically Supported Candidates Awaiting KD Validation

#### 5.5.1. Metformin

Metformin activates AMPK, a master regulator that promotes autophagy and inhibits NF-κB signaling, providing a mechanistic rationale for rebalancing the dysregulated axis extrapolated from its effects in metabolic and cardiovascular disorders [25]. No studies have specifically investigated its role in KD. Therefore, its proposed utility as a metabolic modulator remains purely hypothesis-generating. While extensive pediatric safety data exist for type 2 diabetes, the complete absence of efficacy data in KD models or patients is a fundamental limitation, making it a candidate only for pilot mechanistic studies.

#### 5.5.2. Urolithin A

Urolithin A, a gut microbiome-derived metabolite, enhances mitophagy and inhibits the cGAS-STING pathway, as evidenced by studies in aging and general inflammation models [5,12]. Although there is no direct evidence in KD models, it is a hypothesis-generating candidate for a preventative or disease-modifying role targeting mitochondrial dysfunction. Although its natural origin and initial safety data in adults are encouraging, the critical lack of KD-specific data and high inter-individual variability in its production pose significant barriers to its therapeutic development for KD.

#### 5.5.3. ASK1 Inhibitors (e.g., Selonsertib)

ASK1 inhibitors, such as selonsertib, block the ASK1-p38 MAPK pathway, a stress-activated inflammatory cascade. Indirect mechanistic support comes from a study showing that the autophagy receptor NOD1 degrades ASK1 to suppress coronary inflammation [13], although the inhibitor itself has not been tested in KD. This represents a potential target for mitigating vascular inflammatory signalling. However, development faces challenges including a lack of pediatric KD model data and setbacks in the clinical development of the lead compound for other diseases, placing it at the target validation stage for KD.

### 5.6. Novel Targets at the Discovery Stage

#### FCGR3B-S100A12 Axis Inhibitors

Inhibition of the FCGR3B-S100A12 axis represents a novel, KD-specific strategy targeting a pro-thrombotic and pro-inflammatory pathway identified through in vitro mechanistic studies [37]. It is a discovery-stage target for potential intervention. Although it offers a disease-specific perspective, its causal role in vivo remains unproven, and clinically viable inhibitors do not currently exist, necessitating extensive target validation and drug development efforts before any translational application can be considered.

### 5.7. Integration, Challenges, and Translational Roadmap

In summary, a critical appraisal based on the current evidence hierarchy positions anakinra as the most immediate therapeutic candidate for refractory KD, given its precise mechanism and established pediatric use. Agents with KD-specific preclinical evidence, such as rapamycin, necrosulfonamide, and resveratrol, are promising candidates but require rigorous validation of their safety and, crucially, their feasibility in the context of acute pediatric KD. Strategies involving metformin or urolithin A, although mechanistically supported, remain purely hypothesis-generating for KD and necessitate foundational KD-model studies.

Translational Challenges: Translating therapies targeting the autophagy–inflammation axis into clinical practice for acute KD faces several substantial hurdles. First, the timing of intervention is critical. Autophagy may exert dual, context-dependent roles, and intervening during the peak inflammatory phase versus the convalescent phase could yield vastly different outcomes and risks [5]. Second, there is a pronounced lack of patient-accessible biomarkers. Validated, non-invasive biomarkers for real-time monitoring of autophagic flux or axis activity in patients are unavailable, hindering biomarker-guided patient stratification, personalized dosing, and objective assessment of therapeutic response. Third, pediatric-specific drug development considerations are paramount. Many candidates (e.g., rapamycin, urolithin A) lack pharmacokinetic and safety data for use in acutely febrile children. The poor oral bioavailability of natural compounds (e.g., resveratrol) also limits their efficacy. Fourth, the choice of clinical trial endpoints needs re-evaluation. For drugs aiming to modulate this axis, should traditional endpoints, such as coronary artery lesion incidence, be used, or should surrogate markers reflecting the restoration of cellular homeostasis (e.g., plasma mtDNA levels) be incorporated? This requires a prospective trial design.

A staged translational roadmap is therefore warranted: Near-term (1–3 years): Efforts should focus on optimizing the use of anakinra in refractory KD within controlled clinical trials and validating candidate biomarker panels (e.g., combining mtDNA, inflammasome components, and autophagic markers in PBMCs) for risk stratification of IVIG resistance and CALs. Mid-term (3–5 years): Early-phase clinical trials could evaluate repurposed agents with favorable safety profiles (e.g., bioavailable formulations of resveratrol) in biomarker-defined high-risk subgroups. Research should also optimize the timing and dosing of these interventions within the course of KD. Long-term (>5 years): Development may advance toward advanced therapeutic platforms, including cell-specific targeted delivery systems (e.g., nanocarriers for endothelial or macrophage-specific delivery) and rational combination therapies (e.g., an autophagy inducer with an IL-1β inhibitor), informed by a deeper mechanistic understanding of axis dynamics across disease stages.

**Table 1:** Evaluation of Therapeutic Strategies Targeting the Autophagy–Inflammation Axis in Kawasaki Disease.

Agent/Target	Mechanism of Action (Related to Axis)	Evidence Source and Model	Proposed Therapeutic Objective	Clinical Readiness (KD)	Key Limitations/Safety Considerations
Anakinra	IL-1 receptor antagonist; inhibits NLRP3 inflammasome output.	Clinical evidence: Case series in refractory KD [32,33]; genetic association (IL1B polymorphism) with IVIG resistance [31].	Rescue therapy for IVIG-resistant disease.	Clinical use (refractory cases)	Subcutaneous administration; high cost; need for larger controlled trials.
Sirolimus (Rapamycin)	mTOR inhibitor; induces autophagy; inhibits endothelial inflammation via AKT/mTOR pathway.	Preclinical, KD-specific: In vitro (KD murine model) [34].	Prevention of coronary artery remodeling.	Experimental	Narrow therapeutic window; immunosuppression risk; unclear timing in acute phase.
Necrosulfonamide	Inhibits gasdermin D (GSDMD), blocking pyroptosis and IL-1β release.	Preclinical, KD-specific: In vitro (KD serum-induced pyroptosis in macrophages/HUVECs) [14,15].	Mitigation of acute, severe endothelial inflammation.	Early preclinical	Specificity, pharmacokinetics, and in vivo efficacy not validated; no clinical-grade inhibitors.
Resveratrol	Activates SIRT1/AMPK to enhance autophagy; antioxidant and anti-inflammatory.	Preclinical, KD-specific: In vitro (human coronary artery endothelial cells) [36]; in vivo (KD mouse model) [35].	Adjunctive antioxidant and anti-inflammatory agent.	Experimental/Nutraceutical	Poor oral bioavailability; rapid metabolism; no controlled clinical trials in KD.
Metformin	Activates AMPK, promotes autophagy and inhibits NF-κB signaling.	Mechanistic extrapolation (non-KD): Studies in metabolic/cardiovascular fields [25]. No KD-specific data.	Hypothesis-generating: Metabolic modulator to restore axis balance.	Hypothesis-generating	Complete absence of efficacy and safety data in KD models or patients.
Urolithin A	Enhances mitophagy, inhibits cGAS-STING signaling.	Mechanistic extrapolation (non-KD): Studies in aging and inflammation models [5,12]. No direct KD evidence.	Hypothesis-generating: Preventative or disease-modifying agent targeting mitochondrial dysfunction.	Hypothesis-generating	Lack of KD model data; high inter-individual variation in production.
ASK1 Inhibitors (e.g., Selonsertib)	Inhibits ASK1-p38 MAPK pathway, a stress-activated inflammatory cascade.	Indirect mechanistic support: Study showing NOD1 degrades ASK1 to suppress inflammation [13]. Inhibitor not tested in KD.	Potential target for mitigating vascular inflammatory signaling.	Target validation stage	Lack of pediatric/KD model data; clinical development of lead compound faced challenges.
caspase-1 Inhibitor	Inhibits caspase-1, blocks GSDMD cleavage and IL-1β/IL-18 maturation.	Mechanistic extrapolation (non-KD): Studies in other disease models (e.g., fibrosis) [38]. No KD evidence.	Potential upstream inhibitor of inflammasome activation.	Target validation stage	Absence of KD model data and unproven vascular efficacy in KD context.
FCGR3B-S100A12 Axis Inhibitor	Inhibits a novel pro-thrombotic and pro-inflammatory pathway.	Preclinical, novel KD pathway: In vitro mechanistic study in a KD context [37].	Potential KD-specific target for intervention.	Discovery stage	Causal role in vivo unproven; no clinical inhibitors exist; requires extensive validation.

## 6. Discussion

This review consolidates current evidence to propose that dysregulation of the autophagy–inflammation axis represents a pivotal, although not yet fully validated, mechanism contributing to vascular injury in Kawasaki disease (KD) [1,2,5]. The synthesised model, in which defective mitophagy and lysosomal dysfunction propel inflammation via NLRP3/cGAS-STING, provides a plausible explanation for sustained endothelial damage and aligns with the observed clinical phenotypes [5,9,15]. However, it is crucial to acknowledge that the direct causal link in patients, especially for IVIG non-response, requires further prospective validation. Preclinical investigations have identified promising therapeutic agents targeting this regulatory network; however, critical unresolved controversies and translational barriers persist that must be addressed to bridge the bench-to-bedside gap.

### 6.1. Outstanding Questions and Future Directions

Despite consistent evidence supporting the pathogenic role of an imbalanced autophagy–inflammation axis in KD, several key inconsistencies and knowledge gaps remain, which must guide future research [5].

Validation of Population-Specific and Generalizable Mechanisms. Population-specific pathogenic mechanisms remain incompletely validated in the study. Although MCM8 deficiency has been demonstrated to exacerbate coronary arteritis and activate the cGAS-STING pathway in murine models, the association between the East Asian-enriched MCM8-P276 variant and KD susceptibility raises the possibility of ethnicity-specific pathogenic pathways [9]. This mechanism requires prospective verification in large, multi-ethnic clinical cohorts to determine its global generalizability, which is a prerequisite for developing precision medicine strategies applicable to diverse populations.

Elucidation of Cellular Crosstalk within the Vascular Lesion Microenvironment. Current studies predominantly focus on unidirectional regulatory events—for example, resistin-mediated suppression of endothelial autophagy or macrophage pyroptosis induced by KD serum [14,15,23]—yet the reciprocal modulation of local immune cell function (e.g., CD14+ monocytes and CD19+ B cells) and adaptive immune activation by impaired endothelial autophagy remains poorly defined [8,30]. Elucidating this bidirectional crosstalk is essential for a comprehensive understanding of the pathophysiology of KD vasculitis.

Clarifying the Coordinated Roles of Innate and Adaptive Immunity. The induction of coronary arteritis by pathogen-associated molecular patterns (e.g., CAWS) in mice deficient in adaptive immunity appears inconsistent with clinical observations of aberrant B-cell activation and the therapeutic efficacy of IVIG [7,18]. This discrepancy implies coordinated rather than independent dysregulation of innate and adaptive immune programs in KD, and the underlying integrative mechanisms warrant further mechanistic exploration.

Linking Axis Dysregulation to Clinical Heterogeneity and IVIG Resistance. A major limitation is the weak mechanistic link between autophagy–inflammation axis dysregulation and clinical heterogeneity in KD. Similar degrees of mitophagy suppression are associated with divergent coronary outcomes among individual patients [5,16]. This heterogeneity may be explained by a “second-hit” model, in which genetic susceptibility establishes a baseline autophagic vulnerability that is further exacerbated by environmental triggers, metabolic perturbations, or epigenetic modifications [9,28,29]. Additionally, the causal relationship between autophagy suppression and IVIG non-response remains ambiguous [5]. Clarifying these relationships is critical for accurate risk stratification.

Overcoming Barriers to Therapeutic Translation. Clinical translation faces substantial challenges. Autophagy modulators exhibit a narrow therapeutic window, and the optimal timing of intervention during the acute inflammatory phase remains undetermined. Furthermore, the lack of reliable, non-invasive biomarkers for real-time monitoring of autophagic flux and inflammatory axis activity hinders the implementation of individualised treatment strategies and the assessment of therapeutic efficacy [5].

### 6.2. Translational Roadmap and Clinical Implications

To bridge the gap between mechanistic insights and clinical applications, a staged translational approach is warranted, aligns with the clinical heterogeneity of KD.

In the near term (1–3 years), the most feasible strategy is the repurposing of agents with existing pediatrics safety profiles for severe, IVIG-resistant cases. Anakinra (IL-1 receptor antagonist) is the prime candidate, supported by mechanistic links to NLRP3 overactivation and encouraging clinical case series [31,32,33]. Concurrently, efforts must focus on validating candidate biomarkers derived from this axis (e.g., plasma mtDNA, autophagic flux markers in peripheral blood mononuclear cells) in prospective cohorts to improve risk stratification for IVIG resistance and coronary artery lesions [5,9,16].

In the mid-term (3–5 years), early-phase clinical trials should evaluate novel autophagy modulators with favourable safety profiles (e.g., urolithin A, improved formulations of resveratrol) in high-risk patient subgroups defined by the aforementioned biomarkers [5,12,36]. Research should also optimise the timing and dosing of these interventions within the course of KD disease.

For the long term (>5 years), development should focus on advanced therapeutic platforms. This includes cell-specific targeted delivery systems (e.g., nanocarriers for endothelial or macrophage-specific delivery) and rational combination therapies (e.g., an autophagy inducer with an IL-1 inhibitor) informed by deeper mechanistic understanding. Future research must prioritize the integration of single-cell and spatial multi-omics technologies to characterize the autophagic, metabolic, and inflammatory landscape within vascular lesions, enabling the identification of novel cell-type-specific targets and regulatory networks [8,20].

Ultimately, integrating multi-omics data with clinical phenotypes is essential to transition from current empirical therapy toward pathophysiology-defined precision therapy for KD, with the goal of improving long-term cardiovascular outcomes for all children, especially those resistant to first-line treatments.

## 7. Conclusions

Accumulating evidence suggests that dysregulation of the autophagy–inflammation axis represents a promising pathogenic mechanism underlying KD-related vasculopathy, provides a potential mechanistic link between organelle dysfunction and systemic immune dysregulation, as well as coronary vascular damage. Preclinical data have demonstrated the therapeutic potential of various agents targeting this axis in models, highlighting its value for further exploration as a treatment target for KD.

Nevertheless, critical knowledge gaps persist, including the generalisability of population-specific mechanisms, elucidation of bidirectional cellular crosstalk, and mechanistic relationship between axis dysregulation and clinical heterogeneity. Future investigations employing the integrated, multi-omic approaches outlined above will facilitate the dissection of dynamic regulatory networks within the autophagy–inflammation axis, support the development of clinically applicable risk stratification tools, and enable the design of stage- and cell-specific precision intervention strategies. Translating these research advances into clinical practice has the potential to improve risk assessment, reduce the incidence of coronary complications, and optimise long-term cardiovascular outcomes for children with KD.

## Data Availability

Data sharing not applicable to this article as no datasets were generated or analysed during the current study.
